# Comparative Assessment of Multimodal Sensor Data Quality Collected Using Android and iOS Smartphones in Real-World Settings

**DOI:** 10.3390/s24196246

**Published:** 2024-09-26

**Authors:** Ramzi Halabi, Rahavi Selvarajan, Zixiong Lin, Calvin Herd, Xueying Li, Jana Kabrit, Meghasyam Tummalacherla, Elias Chaibub Neto, Abhishek Pratap

**Affiliations:** 1 Centre for Addiction and Mental Health, Toronto, ON M6J 1H4, Canada; ramzi.halabi@camh.ca (R.H.); rahavi.selvarajan@mail.utoronto.ca (R.S.); zixiong.lin@camh.ca (Z.L.); calvinherd@gmail.com (C.H.); sophiixy@gmail.com (X.L.); jana.kabrit@outlook.com (J.K.); 2 Sage Bionetworks, Seattle, WA 98121, USA; meghasyam@sagebase.org (M.T.); elias.chaibub.neto@sagebase.org (E.C.N.); 3 Department of Psychiatry, University of Toronto, Toronto, ON M5S 1A1, Canada; 4 Vector Institute for Artificial Intelligence, Toronto, ON M5T 1R8, Canada; 5 Institute of Psychiatry, Psychology & Neuroscience, King’s College London, London WC2R 2LS, UK; 6 Department of Biomedical Informatics and Medical Education, University of Washington, Seattle, WA 98195, USA

**Keywords:** digital health, decentralized clinical study, smartphone sensors, digital signal processing, multimodal sensing, data quality, machine learning, model interpretability

## Abstract

Healthcare researchers are increasingly utilizing smartphone sensor data as a scalable and cost-effective approach to studying individualized health-related behaviors in real-world settings. However, to develop reliable and robust digital behavioral signatures that may help in the early prediction of the individualized disease trajectory and future prognosis, there is a critical need to quantify the potential variability that may be present in the underlying sensor data due to variations in the smartphone hardware and software used by large population. Using sensor data collected in real-world settings from 3000 participants’ smartphones for up to 84 days, we compared differences in the completeness, correctness, and consistency of the three most common smartphone sensors—the accelerometer, gyroscope, and GPS— within and across Android and iOS devices. Our findings show considerable variation in sensor data quality within and across Android and iOS devices. Sensor data from iOS devices showed significantly lower levels of anomalous point density (APD) compared to Android across all sensors (*p*  <  1 × 10^−4^). iOS devices showed a considerably lower missing data ratio (MDR) for the accelerometer compared to the GPS data (*p*  <  1 × 10^−4^). Notably, the quality features derived from raw sensor data across devices alone could predict the device type (Android vs. iOS) with an up to 0.98 accuracy 95% CI [0.977, 0.982]. Such significant differences in sensor data quantity and quality gathered from iOS and Android platforms could lead to considerable variation in health-related inference derived from heterogenous consumer-owned smartphones. Our research highlights the importance of assessing, measuring, and adjusting for such critical differences in smartphone sensor-based assessments. Understanding the factors contributing to the variation in sensor data based on daily device usage will help develop reliable, standardized, inclusive, and practically applicable digital behavioral patterns that may be linked to health outcomes in real-world settings.

## 1. Introduction

Smartphones have become an essential part of people’s daily lives, with the projected number of smartphone users reaching 4.74 billion in 2024, a 50% increase in 2018–2024 [1]. Given the ubiquity of consumer-owned smartphones, healthcare researchers are increasingly interested in leveraging smartphones to gather frequent behavioral data within a person’s environment [2] using multimodal onboard sensors [3].

The growing ability to learn personalized health trajectories from real-world data (RWD) [4] gathered actively (e.g., patient self-reported outcomes) and passively (e.g., smartphone/watch-based sensors without active user input) from a large and diverse population is being looked at as a sea change in health research. A decentralized health data collection approach offers a scalable and cost-effective means to frequently monitor health-related behaviors over time sat a fraction of the time and cost; complimenting episodic clinical data collected intraditional health research studies. Clinician assessed outcomes contextualized with RWD may offer a holistic view of individualized health outcomes in real world settings.

High-frequency sensor data collected from smartphones has been used by health researchers to monitor real-world behaviors for example, physical activity [5,6], and physical [7] and mental health [3]. Onboard inertial sensors such as accelerometers, gyroscopes, GPS, etc., are beginning to be used for real-time context analysis such as human activity recognition [5], step counting [8], gait and posture assessment [9], fall detection [10], and sleep patterns [11]. Researchers are also exploring using smartphones to track the heart rate (H.R.) [12], pulse rate (PR) [13], facial expressions [14], skin health [15], and daily mood [16], and predict neurodegenerative diseases early by developing digital markers [17].

However, recent studies have brought several challenges to the surface [18,19] in using smartphones and wearable devices for remote health data collection in real-world settings. In RWD from smartphones. With smartphone ownership in North America distributed closely between iOS (42.0%) and Android (57.6%) [20], health studies may collect variable sensor data from heterogenous devices; impacting the robustness and generalizability of derived digital behavioral signatures. Key factors include differences [21] in hardware and software (e.g., Android vs. iOS) along with variations in device usage by a large population in naturalistic settings [22]. While previous research studies have investigated sensor-specific data quality [23,24,25], the focus has been limited (e.g., missingness of collected data [26]), along with the assumptions of consistent data collection environments and data sampling with uniform noise and anomaly levels.

**Figure 1:**
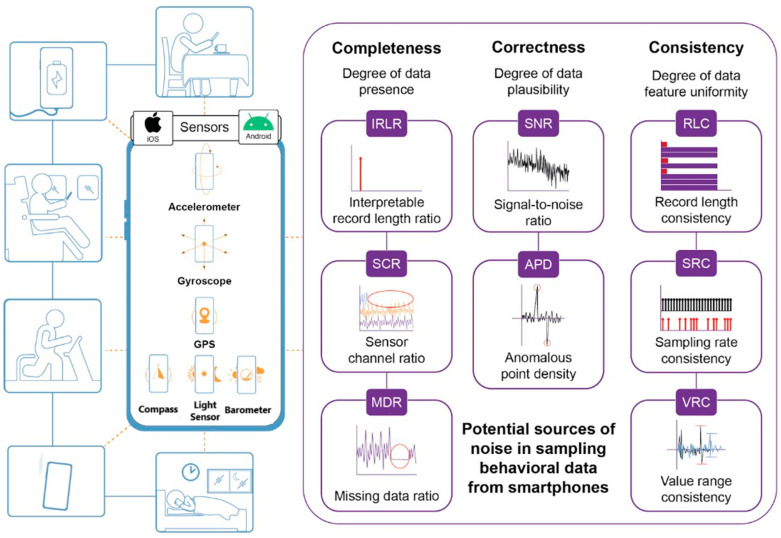
Schematic representation of potential noise sources in collecting smartphone sensor data in real-world settings across three data quality dimensions—completeness, correctness, and consistency. The illustration depicts data quality metrics (DQMs) for a single observation (“record”). The same DQMs can be applied to multiple sensor records to assess temporal variations in sensor data quality within and across study participants.

However, the long-term sensor data collection from smart devices (e.g., smartphones and smartwatches) can vary significantly based on the personalized usage of devices in everyday settings. These include but are not limited to diverse noise sources in sensor data, intermittent loss of coverage, irregular sensor data sampling protocols, and insufficient device battery levels, which may lead to non-stationary time-series data collection [27]. The presence of non-stationarity in medical time series data poses a potential risk to downstream data analysis linked to modelling assuming stationarity data potentially leading to biased inference. These challenges can manifest in inter-device and inter-sensor sampling inconsistency, noise contamination [28,29] (e.g., white noise and baseline wandering), and anomalies (e.g., random spikes and spurious activity) (Figure 1). To address some of these practical challenges in RWD acquisition, the Food and Drug Administration (FDA) has also recently shared guidance for using digital health technologies for remote data acquisition in clinical investigations [30].

To address the potential variation in smartphone sensor data quality in real-world settings, this study conducted a secondary analysis of data collected as part of the Warfighter Analytics using Smartphones for Health (WASH) study [31]. The study used data from 3000 participants enrolled in the WASH study for up to 12 weeks. Of note, the WASH study data collection, including the study app development, was carried out by an independent study team unrelated to this secondary analysis. The two main goals of the present study are to (i) assess the variation in sensor quality data from three specific sensors—the accelerometer, gyroscope, and GPS; and (ii) evaluate the inter-device (Android vs. iOS) and inter-sensor (accelerometer vs. gyroscope vs. GPS) variation in data quality. Additionally, we used a machine learning approach to evaluate if sensor data quality metrics alone could predict device type (Android vs. iOS). This is to assess the extent to which data quality differences between Android and iOS devices could potentially confound behavioral trajectory or disease severity prediction.

## 2. Materials and Methods

### 2.1. Study Summary

The present study conducted an independent secondary analysis of the smartphone sensor data collected in the WASH study [32]. The original research, funded by the United States Air Force and DARPA, aimed to assess real-world factors that could help predict cold and flu early and examine the impact of traumatic brain injury on daily functioning [31]. The research aimed to understand novel real-world factors that could help assess the readiness of warfighter veterans. The enrollment was open to all US residents, meeting the inclusion/exclusion criteria. Eligible participants gave their consent electronically and shared their data by downloading the study app on their smartphones, which was available on iOS and Android. The participants in the WASH study were US volunteers who agreed to participate in a 12-week smartphone-based study. The University of Washington’s Institutional Review Board and the Department of Defense Human Research Protection Office (HRPO) approved the study.

The availability of large-scale raw sensor-level data provided an ample dataset for comparison of sensor data within and across Android and iOS devices. Of note, the data quality analysis was conducted independently of the study app developer.

### 2.2. Data Collection

Participants in the study enrolled remotely through a study portal. After enrollment, participants completed questionnaires (e.g., sociodemographics and health-related surveys) and were guided to install the study app (HIPPOCRATIC App) on their smartphones (see Appendix A). The study app was developed by an independent company, Kryptowire LLC (McLean, VA, USA).

The app was developed as a native application and is available for iOS and Android platforms. Consenting participants shared sensor data from their smartphones’ (Android or iOS) data throughout the study observation period (up to 12 weeks) using the study app. The app collected raw sensor data directly from onboard smartphone sensors such as accelerometers, gyroscopes, and GPS through the native operating system’s APIs. The native access enabled the study app to collect high-fidelity sensor data with minimal preprocessing such that the raw sensor data are transmitted with minimal alteration by the mobile OS or the app itself, providing a reliable dataset for subsequent quality analysis. The study app on the Android platform allowed participants to share more passive sensor data types (up to 21). At the same time, the iOS version collected data from up to 7 sensors (see Supplementary Table S1 for further details). This significant difference in the number of sensor data streams available between Android and iOS is due to differences in data-sharing restrictions and a broader array of composite sensors available amongst Android phones.

More than 30 terabytes of sensor-based passive data were collected from study participants. Due to raw sensor data size, accessibility, and data download constraints, we used sensor data from a subset of study participants (N = 3000) for the present analysis. The participants in the current study cohort were selected to broadly represent the overall study cohort, balancing user device types (i.e., Android and iPhone) and participant engagement. In addition, a subset of study participants identified by the WASH study team as malicious actors not meeting the inclusion–exclusion study criteria was removed from the analysis cohort [32]. Supplementary Table S2 compares the sociodemographics of the selected cohort and the entire study cohort.

### 2.3. Sensor Data Quality Metrics

In designing the sensor data quality metrics (DQMs), we accounted for operating system (OS)-specific factors that could influence the quality and consistency of sensor data collected from iOS and Android devices. Known differences such as power-saving strategies, latency, and sensor interfacing were considered to ensure DQMs could robustly capture and quantify such differences across different platforms. For instance, iOS emphasizes user privacy with granular permission systems and App Tracking Transparency, which can impact data collection consistency, particularly for location services. These features may contribute to higher missingness in GPS data from iOS devices than Android. Smartphone sensor data quality was assessed across three main dimensions—completeness, correctness, and consistency—using eight data quality metrics (DQM) described below and illustrated in Figure 1. We used the median value of each DQM per sensor for an individual participant’s sensor data collected during the observation period (up to 12 weeks) for data quality analysis. Each short duration of continuously sampled passive data observation is called a “record”. The mathematical definition of each DQM is described below and summarized in Table 1.

#### 2.3.1. Completeness

Sensor data completeness is the extent to which the sensor data are collected or missing for each participant’s individual observation period. Three DQMs were developed to assess data completeness and were scaled using min–max scaling [31].

(i.) Interpretable record length ratio (IRLR): the ratio of the number of records having two or more data points over the total number of records, calculated as in (1):

(1) $$IRLR=\frac{n(len>1)}{N}\;,$$

where *n*(*len* > 1) is the number of records having a length of 2 points and above, and *N* is the total number of records. *IRLR* can be used to investigate a commonly observed quality issue that arises when the data collection app creates and uploads a record file that includes no data points or a single uninterpretable point.

(ii.) Sensor channel ratio (SCR): ratio of available channels per record by the typical number of channels for each sensor, calculated as in (2) and (3):

(2) $$SCR=\frac{SCC}{ESCC}\;,$$

(3) $$ESCC=mode\left(SCC\right)\;,$$

where *SCC* is the sensor channel count, and *ESCC* is the expected sensor channel count calculated as the most frequent channel count per sensor. SCR allows the detection of missing channels (e.g., acceleration across the z-axis) in case of sensor defect or partial data upload.

(iii.) Missing data ratio (MDR): ratio of missing data points over the total number of record points for all sensor types. The MDR metric measures the level of discontinuity manifested through skipped data points due to under-sampling, calculated as in (4):

(4) $$MDR=\frac{MPC}{TPC}\;,$$

where *MPC* is the missing point count, and *TPC* is the total point count, calculated as in (5) and (6):

(5) $$MPC+=ceil\left(\frac{t\left(n+1\right)-t\left(n\right)}{\hat{t_{s}}}\right)-1,\;if:t\left(n+1\right)-t\left(n\right)>\hat{t_{s}}\;,$$

(6) $$\hat{t_{s}}=mode\left(t_{s}\right)\;,$$

where $ceil\left(.\right)$ is a function that returns the integer greater than or equal to the input number, *t*(*n*) is the timestamp of point *n*, $t_{s}$ is the sampling interval, and $\hat{t_{s}}$ is the data-driven sampling interval calculated as the most frequent time interval. This type of data missingness quantifies the level of information loss, guiding the need for appropriate data resampling and interpolation methods.

#### 2.3.2. Correctness

Sensor data correctness is the level of plausibility of a data record’s values and structure during observation. Two DQMs were developed to assess data correctness and scaled via min–max scaling:

(i.) Signal-to-noise ratio (SNR): amplitude or power ratio of the signal component, i.e., desired data by the noise component. *SNR* describes the level of data variability around the mean absolute value, approximating the proportion of noise contamination with respect to the desired data of interest, calculated as in (7)–(9):

(7) $$SNR=20\cdot log\left(\frac{S}{N}\right)$$

(8) $$S=MAV\left(x\right)$$

(9) $$N=\sigma_{x}$$

where *S* is the desired data (signal) approximated as the *MAV* (mean absolute value) of data record *x*, and *N* is the undesired data (noise) approximated as the standard deviation of data record *x*. This metric gives insight into the noise level rather than its type, providing feedback on the required level of data denoising.

(ii.) Anomalous point density (APD): ratio of anomalous points by the total number of recorded points, calculated as in (10) and (11):

(10) $$APD=\frac{APC}{TPC\;}$$

(11) $$APC+=1,\;if:DS\left(x\left[n\right]\right)>\bar{DS\left(x\right)}+3\cdot \sigma_{DS\left(x\right)},$$

where APC is the anomalous point count, and TPC is the total point count. The APC is incremented by 1 whenever the decision score (*DS*) of a data sample *x*[*n*] exceeds the set threshold being a z-score of 3 [32]. The APD metric allows for detecting and counting outliers in sensor data on a point-by-point basis, resulting in a density estimate of anomalies. It is computed using an unsupervised machine learning technique termed feature bagging method [33], followed by outlier score thresholding [34] to count anomalous points.

#### 2.3.3. Consistency

Sensor data consistency is the degree of invariance in one or more data records. Three DQMs were developed to assess data consistency. The consistency metrics were computed by constraining the inverse of the coefficient of variation [35] via a sigmoid-based function such that very high values are set to 1, indicating near-zero variance referred to as a “normalized reciprocal”.

(i.) Record length consistency (RLC): measure of uniformity between the lengths of different data records across sensors. It is the inverse of the coefficient of variation of record length variability across data records, where record length is the number of points per record. RLC is calculated as in (12):

(12) $$RLC=2\left(1-sig\left(\frac{\sigma_{RL}}{\bar{RL}}\right)\right),$$

where *sig*(.) is the sigmoid function, $\sigma_{RL}$ is the standard deviation of record lengths, and $\bar{RL}$ is the mean record length. The higher the RLC is, the less the burden of data segmentation and preprocessing is before the analysis.

(ii.) Sampling rate consistency (SRC): inverse of the coefficient of variation of the sampling intervals across sensors with the sampling interval as the difference between two consecutive data point timestamps, calculated as in (13):

(13) $$SRC=2\left(1-sig\left(\frac{\sigma_{t_{s}}}{\bar{t_{s}}}\right)\right),$$

where *sig*(.) is the sigmoid function, $\sigma_{t_{s}}$ is the standard deviation of sampling intervals, and $\bar{t_{s}}$ is the mean sampling interval. The higher the SRC is, the more regular the data sampling is.

(iii.) Value range consistency (VRC): inverse of the coefficient of variation of record data value min–max range variability across sensors with the min–max range describing the difference between the maximum and minimum value of a record. VRC is the degree of stability of the data value range across sensors, calculated as in (14):

(14) $$VRC=2\left(1-sig\left(\frac{\sigma_{VR}}{\bar{VR}}\right)\right),$$

where *sig*(.) is the sigmoid function, $\sigma_{VR}$ is the standard deviation of value ranges, and $\bar{VR}$ is the mean value range.

Higher VRC indicates lower range variability, hence higher reproducibility and sensor output stability. All three consistency DQMs typically require more than one data record for accurate computation and are otherwise set to 1 for a single record, except for SRC, which computes intra-record consistency.

To enable cross-comparison of DQMs within and across devices, each of the eight DQMs was normalized across sensors and participants except for the SNR DQM to preserve the standard scale and unit. This allowed for data-driven data quality assessment independently of sensor type, device type, and inter-participant differences, enabling the reusability of the DQMs for any smartphone sensor dataset requiring no calibration or tuning.

### 2.4. Sensor Data Quality Analysis

#### 2.4.1. Data Featurization

To assess cross-sectional differences in sensor data quality, the sensor data collected longitudinally from participants during the observation period (up to 12 weeks) were used to generate the average DQM value per sensor per participant. To quantify the differences in sensor data quality, eight DQMs (described above) were generated per participant for each of the three sensors—accelerometer, gyroscope, and GPS. The data record count distribution across devices and sensors is presented in Supplementary Table S7.

#### 2.4.2. Statistical Analysis

Cohort-level descriptive statistics were generated for each DQM, stratified by device type (Android vs. iOS) and sensor type (accelerometer, gyroscope, and GPS). We used non-parametric Mann–Whitney (MW) to assess the significance levels of statistical differences in the underlying non-parametric DQM distributions across devices, sensors, and sub-cohorts. Additionally, we compared potential differences in the cohort’s sociodemographic characteristics between Android and iOS users.

To test the association between DQMs and cohort sociodemographic, we used the Kruskal–Wallis (KW) test followed by the post hoc Dunn test. *p*-values were corrected for multiple testing by controlling the False Discovery Rate (FDR) using the Benjamini–Hochberg method [36]. The pairwise effect size ε² [37] of sociodemographic features on sensor data quality was computed and filtered based on statistical significance. Statistical analyses were conducted using Python version 3.8, and statistical significance was assumed for q-values (FDR-corrected *p*-values) < 0.05.

#### 2.4.3. Device Classification Using Sensor Data

We utilized a machine learning approach to demonstrate the practical implications of variations in data quality between iOS and Android platforms. Our goal was to determine if the sensor data alone could accurately predict the device type. If this is the case, the underlying differences in sensor data could potentially confound the comparison and standardization of disease progression or severity prediction across diverse devices within a large population.

An ensemble-based supervised machine learning approach (Random Forest [38]) was used to investigate the predictive ability of sensor DQMs to classify device types. Device type labels were binary-encoded as (Android = 0, iOS = 1). Input data were split into training and test sets via a 70/30 shuffle split stratified by device type. We performed hyperparameter tuning on the training set using stratified 12-fold cross-validation with random shuffling to select the following parameters: max depth, number of trees, and minimum sample leaf. Stratified k-fold method [39] was selected to account for class imbalance in device types. We evaluated the model’s performance for accuracy, precision, and recall for every parameter combination at the optimal thresholds. The threshold that yielded the highest Youden’s J-statistic [40] was selected for the area under the receiver operator curve (AU-ROC), and the one that yielded the highest F-score was selected for the area under the precision–recall curve (AU-PRC). The model hyperparameters with the highest performance across all three metrics—Accuracy, AU-ROC, and AU-PRC—across the 12 folds were selected to train the model on the training set. We reported the model performance metrics on the held-out test set. We also evaluated the sensitivity of model predictions by using a permutation analysis approach [41]. The device labels were randomly shuffled 1000 times to generate a distribution of model performance metrics for the null hypothesis; i.e., there is no difference in DQMs across device types.

To interpret the contribution of specific DQMs to the classification model performance, we used SHapley Additive exPlanations (SHAP) [42]. SHAP is an algorithm that combines game theory with local explanations and allocates an impact score on classification output for each input feature. We assessed the importance of each feature by computing the importance score (IS) as the absolute average SHAP value. The IS is a positive value (0 ≤ IS ≤ 1) representing the overall predictive power of each DQM to the classification model’s output. For assessing the directional contribution of a feature, the impact level (IL) of each DQM was computed. The IL values vary between −1 to 1, and their sign represents class-specific orientation, with the value representing the relative predictive power. In this study, a negative IL value indicates DQM value to be closer to Android data, while a positive IL is oriented towards iOS. An ideally impactful DQM would have symmetrically bidirectional IL distribution, such that lower values tend towards one class, and higher values tend towards the other.

## 3. Results

### 3.1. Cohort Characteristics

The race/ethnicity of the cohort (N = 3000) was diverse, with the majority of the participants self-reporting as Non-Hispanic white (51.8%, n = 1204), followed by Asians (15.7%, n = 365) and Hispanic/Latinos (13.6%, n = 316). Most participants were females (53.7%, n = 1226), with a higher proportion of younger participants (19–29 years = 49.7%; n = 1039), and with those 60 years and older being the smallest group (4.9%, n = 103).

Participants’ characteristics varied notably between iOS and Android users in the study (See Table 2). The proportion of Black/African-American participants using iPhones was significantly lower than Android (iPhones = 6.2%, n = 71/1145; Android 18.1%, n = 215/1194; *p*-value < 0.001). A significantly higher proportion of iPhone users reported having attained college or graduate school (92.4%, n = 1051/1137) compared to Android (74.0%, n = 876/1184) (*p*-value < 0.001). Additionally, a larger proportion of iPhone users were younger between 19–25 years (iPhone = 56.1%, n = 617/967) compared to Android users (42.5%, n = 422/993) (*p*-value < 0.05). And, finally, participant-reported income levels varied across iPhone and Android users. Notably, the income levels of iPhone users in the study cohort were skewed, with a considerable proportion having an income of less than $25,000 (33.7%, n = 379/1125) compared to Android users (24.5%, n = 237/967) (*p* < 0.05).

### 3.2. Sensor Data Quality Metrics

#### 3.2.1. Overall Data Quality

Sensor data quality was evaluated across three dimensions—completeness, correctness, and consistency—with a total of eight data quality metrics (DQMs). The completeness and consistency of DQMs were reported as unsigned ratios, i.e., between 0 and 1, while the signal-to-noise ratio (SNR) correctness was represented in standard signed dB units, such that a negative SNR indicates more noise than signal (see Methods). In terms of completeness, the overall sensor data quality showed high data point missingness (Median (IQR) = 0.30 (0.42)). As for data correctness, anomalous point density levels (Median (IQR) = 0.014 (0.008)) were low with a negative signal-to-noise ratio (SNR) (Median (IQR) = −3.96 (5.10) dB). The assessment of data consistency showed high inconsistencies in the sampling rate (Median (IQR) = 0.38 (0.39)), record length (Median (IQR) = 0.55 (0.22)), and value range (Median (IQR) = 0.66 (0.28)). No significant association between DQMs and the cohort sociodemographic was seen except for data point missingness, which was statistically significantly associated (*p* < 0.01) with race, specifically for Black/African-American study participants using Android devices (see Figure S1, and Tables S3 and S4 for further details).

#### 3.2.2. Data Quality Differences between Android vs. iOS Devices

The data quality metrics for sensor data varied significantly between iOS and Android smartphones. From a data completeness perspective, missing data ratio levels were significantly higher in Android data (Median (IQR) = 0.39 (0.38)) versus iOS data (Median (IQR) = 0.16 (0.36)) (*p* < 0.0001). However, despite the lower missingness level in iOS data, the GPS data in iOS were characterized by a high missing data ratio (MDR) (Median (IQR) = 0.99 (0.06)) compared to Android (Median (IQR) = 0.42 (0.38)) (*p* < 0.001).

**Figure 2:**
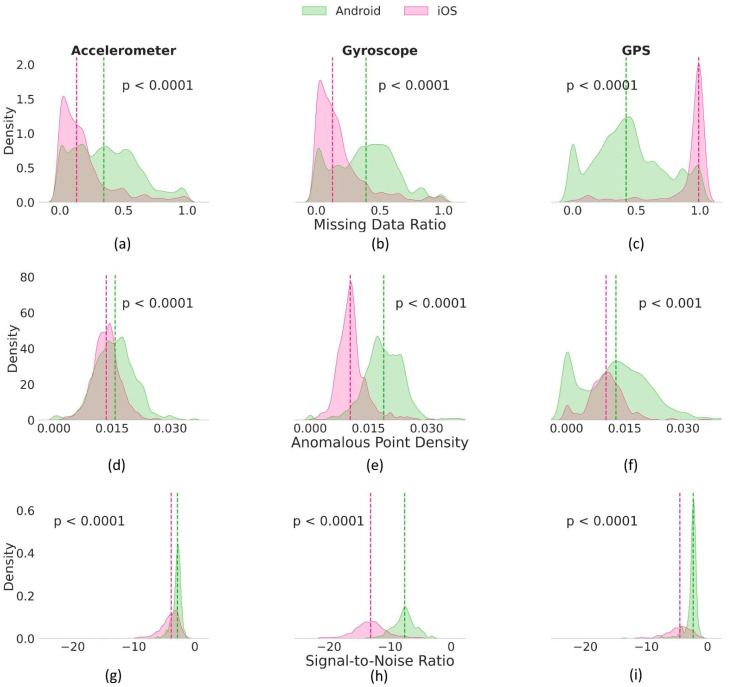
Density histograms comparing selected data quality metrics (DQMs) across three sensors—Accelerometer, Gyroscope, and GPS—stratified by the device (Android in green, iOS in pink). Device-specific medians for each DQM are shown as vertical dashed lines. Significant differences (*p* < 1 × 10^−4^) in data point missingness across device types: (**a**) Accelerometer (iOS: Median = 0.13 vs. Android: Median = 0.34), (**b**) Gyroscope (iOS: Median = 0.13 vs. Android: Median = 0.39), and (**c**) GPS data (iOS: Median = 0.99 vs. Android: Median = 0.42). Data point anomaly levels showing significant differences (*p* < 1 × 10^−3^) in (**d**) Accelerometer (iOS: Median = 0.01 vs. Android: Median = 0.02), (**e**) Gyroscope (iOS: Median = 0.01 vs. Android: Median = 0.02), and (**f**) GPS data (iOS: Median = 0.01 vs. Android: Median = 0.02). The signal-to-noise ratio (SNR) varied significantly (*p* < 1 × 10^−4^), with SNR for iOS being lower: (**g**) Accelerometer (iOS: Median = −3.88 dB vs. Android: Median = −2.85 dB), (**h**) Gyroscope (iOS: Median = −13.2 dB vs. Android: Median = −7.63 dB), and (**i**) GPS data (iOS: Median = −4.5 dB vs. Android: Median = −2.35 dB).

As for sensor data correctness, iOS data were characterized by low anomalous point density (APD) levels compared to Android (Median (IQR): iOS = 0.011 (0.005), Android = 0.016 (0.008)) (*p* < 0.0001). The overall signal-to-noise ratio (SNR) for sensor data collected from iOS devices was lower compared to Android (Median (IQR): iOS = −5.73 (8.86) dB, Android = −3.01 (3.77) dB)) (*p* < 0.0001). In terms of data consistency, a distinct difference in data value range and the sampling rate was observed between the data collected from Android and iOS. The value range consistency (VRC) in iOS (Median (IQR) = 0.73 (0.16)) was higher than that of Android data (Median (IQR) = 0.61 (0.34)) (*p* < 0.0001). Similarly, the iOS sampling rate consistency (Median (IQR) = 0.42 (0.42)) was significantly higher compared to Android (Median (IQR) = 0.35 (0.35)) (*p* < 0.0001). The device-specific DQM statistics are presented in Supplementary Table S5, and the distribution of the most significant DQMs per device type is illustrated in Figure 2. See Table S6 for more details on the overall pairwise comparison of all DQMs across sensors.

#### 3.2.3. Device Type Prediction Using Sensor DQMs

To assess the underlying differences in sensor data gathered from iOS and Android devices, we used an ensemble-based machine learning approach (Random Forest) [38] to predict the device type (Android vs. iOS). The eight DQMs (see Table 1) were used as features. Two different device classification approaches were used: (a) a combined model integrating sensor DQMs across three sensors to classify the device type, and (b) individual sensor-specific models to classify the device type.

**Figure 3:**
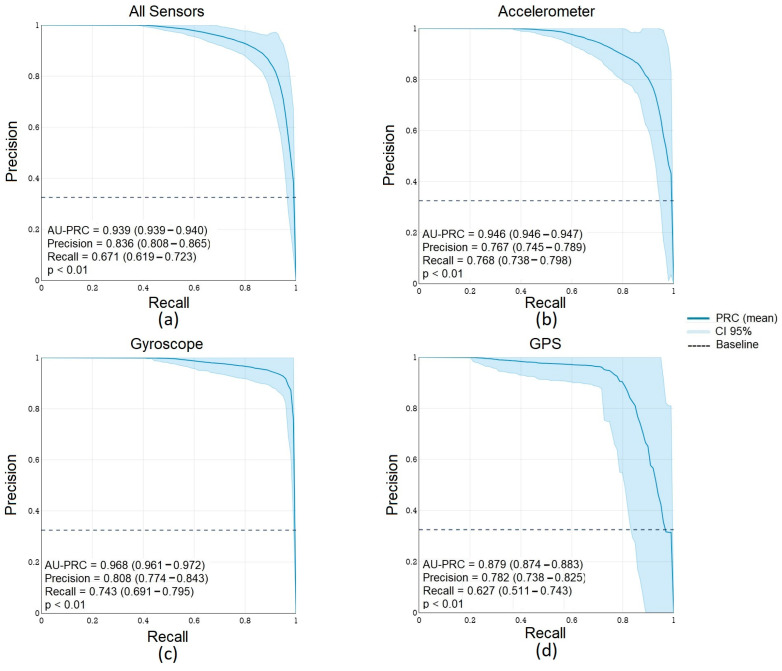
Overall and sensor-specific device type classification precision–recall curves (PRCs). Median PRC bounded by 95% CI for 1000 stratified 12-fold cross-validation permutations using (**a**) overall sensor DQMs; (**b**) accelerometer-specific DQMs; (**c**) gyroscope-specific DQMs; and (**d**) GPS-specific DQMs.

The combined classification model using DQMs for all three sensors showed a high classification accuracy (0.906, 95% CI [0.905, 0.907]). The individual model using gyroscope-derived DQMs showed the highest accuracy (0.979, 95% CI [0.977, 0.982]), followed by the model using the accelerometer (Accuracy = 0.946, 95% CI [0.941, 0.954] and GPS (Accuracy = 0.868, 95% CI [0.861, 0.872] (Table 3). The overall and sensor-specific precision–recall curves and model assessment metrics are illustrated in Figure 3. In addition, see Figure S2 for sensor-specific ROC curves and metrics. The model sensitivity assessment using permutation analysis is presented in Figure S3.

#### 3.2.4. Key DQMs Driving Android vs. iOS Prediction

To help interpret the underlying differences driving the variation of DQMs from same-sensor data between Android and iOS, we used the normalized SHAP importance score (IS) and impact level (IL) metrics [33]. Briefly, the IS metric quantifies the contribution of each input feature (DQM) on model prediction (0: no impact, 1: maximum impact). The SHAP IL values help interpret the influence of the DQM distribution on prediction (iOS: 0  ≤  IL  ≤  1; Android: −1  ≤  IL  <  0). Additionally, the symmetry of the IL values’ distribution for a feature indicates the importance of a bidirectional feature for classifying Android and iOS devices. Here, we report the median values for th eDQM importance scores (IS) and levels (IL) (see Methods for further details). Here, we report the median IS and IL values.

**Figure 4:**
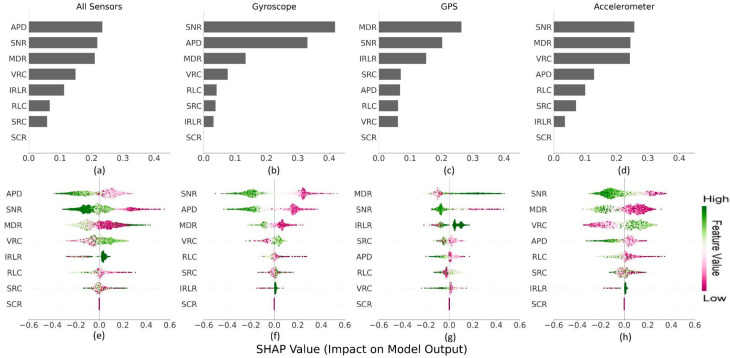
DQM feature importance scores and impact level plots for the combined sensors and sensor-specific DQM impact on device type classification; (**a**–**d**): mean DQM feature importance levels on the combined and sensor-specific model outputs: APD: anomalous point density; SNR: signal-to-noise ratio; MDR: missing data ratio, VRC: value range consistency; IRLR: interpretable record length ratio; RLC: record length consistency, SRC: sampling rate consistency, and SCR: sensor channel ratio; (**e**–**h**): bidirectional DQM SHAP impact levels on combined and sensor-specific model outputs.

In the combined model using summary DQM values for all three sensors, the anomalous point density (APD) had the highest feature importance in device type classification (IS = 0.23), followed by the SNR (IS = 0.22) and MDR (IS = 0.21) (Figure 4a). However, the influence of DQM values (indicated by IL values) on model prediction varied: (i) IL for anomalous point density feature (APD) was asymmetrically bidirectional (i.e., unevenly predictive of device type) and had a moderate impact on device type classification (IL_Android_ = −0.4; IL_iOS_ = 0.3); (ii) IL values for the signal-to-noise ratio (SNR) metric were asymmetrically bidirectional, with the lower SNR values observed in the iOS data associated with stronger IL scores (IL_iOS_ = 0.6) compared to Android (IL_Android_ = −0.3); and (iii) the missing data ratio (MDR) showed a strong and unidirectional impact on device classification (IL_Android_ = −0.4; IL_iOS_ = 0.2) (Figure 4e). The high IL scores for iOS indicate a strong impact of the SNR feature on device type classification and that the classifier tended to leverage the lower SNR values observed in the iOS data more strongly when predicting device type.

The top DQMs from individual sensor-specific models for device classification also showed variations in features that were most predictive of Android and iOS devices (Figure 4a). The SNR and APD DQMs for gyroscope data were highly discriminative of device types (IS_SNR_ = 0.45 and IS_APD_ = 0.35) (Figure 4b). In terms of IL values, SNR IL was symmetrically bidirectional and strongly impacted the device type classification (IL_Android_ = −0.6; IL_iOS_ = 0.6). Figure 4f shows that the classifier using gyroscope data tended to leverage the low SNR values in iOS and the high SNR values in Android data similarly when making predictions.

For GPS, the two DQMs, MDR and SNR, showed high inter-device predictive power (IS_MDR_ = 0.25; IS_SNR_ = 0.22) (Figure 4c). Figure 4g shows that the IL distribution for the MDR was asymmetrically unidirectional towards iOS, with a high impact on device classification (IL_iOS_ = 0.55). This indicates that the classifier tended to leverage, to a greater extent, the remarkably high missingness observed in the iOS GPS data compared to Android data (IL_Android_ = −0.2). Figure 4g also shows an asymmetrically unidirectional distribution of IL values for the SNR DQM from GPS, with the low SNR observed in iOS data leading to a much more substantial impact on device classification (IL_iOS_ = 0.55) when compared to the higher SNR values observed in Android (IL_Android_ = −0.15).

Lastly, Figure 4h shows that the IL scores for the VRC DQM were asymmetrically bidirectional with lower consistency values observed in the Android accelerometer data associated with more substantial IL scores (IL_Android_ = −0.35) in comparison to iOS (IL_iOS_ = 0.25). This indicates that the classifier tended to leverage the lower consistency observed in the Android data (relative to the iOS data) when making the predictions.

#### 3.2.5. Inter-Sensor Data Quality Differences—Accelerometer vs. Gyroscope vs. GPS

Comparing normalized DQMs across the three sensors showed significant data quality differences. Gyroscope data showed high noise levels (SNR: Median(IQR) = −9.74(5.70) dB) in comparison to accelerometer and GPS data (Median(IQR)—accelerometer = −3.11(1.38) dB); GPS = −2.52(1.45) dB) (*p* < 0.0001). However, the proportion of anomalies in the sensor data (indicated by APD) in GPS data (Median(IQR) = 0.012(0.011)) was lower than that of the accelerometer data (Median(IQR) = 0.014(0.006)) and gyroscope data (Median(IQR) = 0.014(0.009)) (*p* < 0.0001). On the data completeness level, the GPS had the highest missing data ratio (MDR) across device types (Median(IQR) = 0.51(0.64)) compared to the gyroscope (0.23(0.37)), and the accelerometer (0.22(0.37)) (*p* < 0.0001). This level of inter-sensor quality difference in the GPS MDR was mainly driven by inter-device differences (Median (IQR)—Android = 0.42(0.38) vs. iOS = 0.99(0.06); *p* < 0.0001).

**Figure 5:**
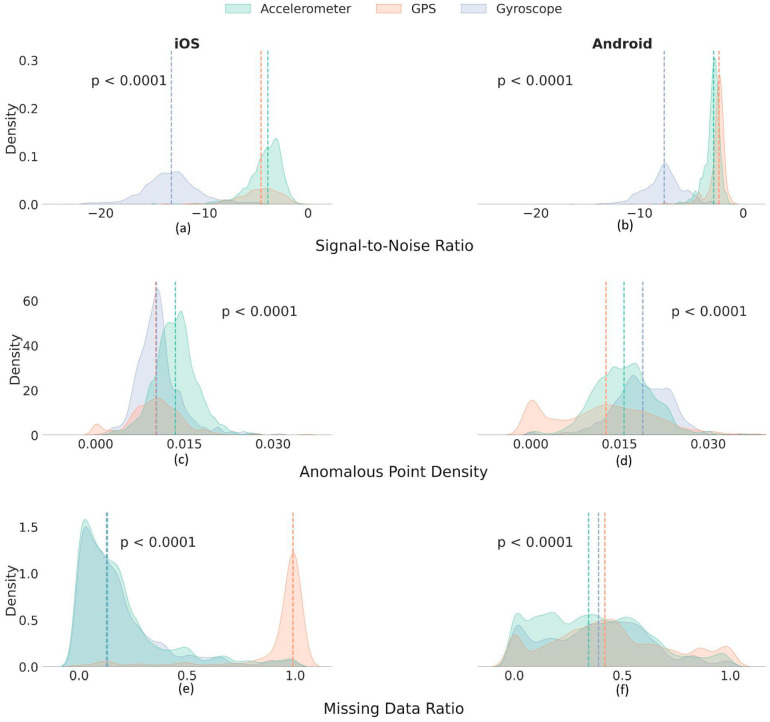
Density histograms for selected data quality metrics across device types—Android and iOS—stratified by the sensor (accelerometer in green, GPS in orange, and gyroscope in blue). Sensor-specific medians for each DQM are shown as vertical dashed lines; (**a**,**b**) density plots showing significant differences (*p* < 1 × 10^−4^) in data noise levels across sensors, with the lowest SNR scored by gyroscope data for both Android (Median = −7.6 dB) and iOS (Median = −13.1 dB); (**c**,**d**) density plots showing significant differences (*p* < 1 × 10^−4^) in data point anomaly levels. GPS data showed lower anomalous point density (APD) across sensors. GPS data scored the lowest APD levels for both Android (Median = 0.013) and iOS (Median = 0.010); (**e**,**f**) density plots showing significant differences (*p* < 1 × 10^−4^) in data point missingness across sensors. GPS data showed the highest MDR, most remarkable in iOS (Median = 0.99), while accelerometer data showed lower MDR levels across Android (Median = 0.34) and iOS (Median = 0.13).

Sensor data consistency also varied significantly between sensors. The value range was least consistent for GPS data (Median(IQR) = 0.42(0.52)) compared to the gyroscope (0.70(0.20)) and accelerometer (0.69(0.19)) (*p* < 0.0001). Similarly, GPS sensor data showed the lowest levels of sampling rate consistency (SRC) (Median(IQR) = 0.30(0.43)) compared to the accelerometer (0.42(0.40)) and gyroscope data (0.40(0.43) (*p* < 0.0001). Figure 5 compares selected DQM distributions across the three sensors faceted by device types. See Figures S4 and S5 for complete DQM distributions across all data quality dimensions.

## 4. Discussion

Our research findings, analyzing one of the largest smartphone sensor datasets available, revealed a significant variation in the quality of sensor data collected in real-world settings within the WASH study population. Here, we outline the main findings and provide a broader perspective to help inform the collection and analysis of multimodal sensor data in large healthcare research studies.

The combined multi-sensor data (accelerometer, gyroscope, and GPS) in the present study had lower overall data missingness for iOS than for Android devices. However, iOS GPS data had exceptionally higher missingness than Android GPS data. This large data missingness gap is mainly due to device-specific GPS sampling irregularities in the present dataset being notably higher in iOS devices. In fact, Apple’s iOS platform strongly emphasizes user privacy, requiring apps to obtain explicit user consent before accessing location services [43]. This is facilitated through a granular permissions system and regular reminders for users to review which apps can access their location data. Furthermore, Apple has introduced the App Tracking Transparency framework, which necessitates apps to secure user permission before tracking their activities across other companies’ apps and websites for advertising or data brokers [44]. The iOS and Android devices showed variability across the levels of noise trends and point anomalies in the data. The data collected using Android devices showed less noise contamination across sensors, although accompanied by more frequent anomalies, compared to iOS data. Lastly, the sensor data acquired using iOS devices were notably more consistent with sampling rates and value ranges. In the present study, the participant sociodemographic had minimal influence on sensor data quality.

With smartphone ownership in North America distributed closely across iOS (42.0%) and Android (57.6%) [20], large remote studies using heterogenous devices [45] will encounter a higher variation in sensor data quality.. High degree of variation in sensor data quality will ultimately impact the accuracy and robustness of digital measures and their relation to of clinical outcomes of interest. Generating robust, representative digital behavioral signatures using sensor data may critically depends on data transformation and featurization, e.g., the mean absolute value, frequency, and dynamic range. Hence, inter-device variations in data completeness may cause an imbalance and potentially representational bias towards participants using devices likely to generate more complete data. For instance, a decentralized study gathering location data through smartphones [46] could have a significant data imbalance across device types, such that the GPS data from Android users largely exceeds iOS data. Moreover, it is essential to note that missingness in sensor data may not be fully resolved or mitigated by traditional resampling methods [47], requiring adaptive multivariate trend modeling and interpolation via pattern recognition [48]. Such imputation approaches may discard data records with higher point missingness levels, uninterpretable length, or missingness in sensor data channels, creating a considerable data imbalance across device types.

In fact, the high accuracy in classification of devices (Android vs. iOS) based on DQMs alone in the present study illustrates how variations in sensor data quality can potentially introduce significant biases into downstream inference derivation. With research studies increasingly using mixed digital devices for disease phenotyping (e.g., rheumatoid arthritis [49] and multiple sclerosis [50]), there is a risk of skewed predictions when comparing data from devices with inherent variability. These challenges are critical to consider, as they could impact the consistency and accuracy of digital measures collected at scale in comparison to clinically outcomes of interest.

Furthermore, inter-device differences in data correctness, such as anomalies and noise patterns, can lead to potentially erroneous digital signatures that may not represent the underlying clinical symptomatology. For instance, the sensor data collected from iOS devices in the present study were more likely to be accurately collected and interpreted. Therefore, the inference drawn using machine learning methods on such sensor datasets could be biased towards iOS devices and that of the population using iOS devices. Unless the entire dataset is screened for anomalies and noise, machine learning models could overfit such noise trends, generating inaccurate feature differences driven by technical differences (e.g., different devices) [51]. And, finally, studies using spectral features to generate digital biomarkers [52] that assume a constant sampling rate for the frequency approximation can be significantly impacted by inconsistencies in sensor data sampling rates.

Missingness in sensor data is mainly driven by sampling discontinuities and irregularities that may also be linked to the day-to-day variation in individuals’ naturalistic environment. This may be further explained by systematic study-related issues such as data collection settings and restrictions imposed by the iOS and Android operating systems [53]. The level of data missingness is also dependent on device- and app-specific data collection protocols during periods of no engagement. In fact, battery-intensive sensors (e.g., GPS) are controlled in a need-based on/off control algorithm to optimize power and computational efficiency, causing the intermittent loss of passive location data collection data missingness. This was highlighted by Currey et al. [54] in a retrospective review of smartphone application digital phenotyping studies, including 1179 participants diagnosed with schizophrenia, anxiety, and depression, reporting a 19% increase in GPS and accelerometer data missingness after only three days of no engagement. Furthermore, unobserved individual behavior (e.g., the sensor on/off control, phone charging, and GPS coverage) are major factors that may have caused sensor data acquisition interruption.

Moreover, inconsistently sampled sensor data could be due to several factors, e.g., sensor dysfunction, device latency due to battery and memory management, storage being full or damaged, and app or operating system settings/constraints. Although a high MDR in real-world sensor data may not be completely avoidable, potential solutions such as weighted resampling [55], sparse online Gaussian process [56], deep regularization techniques [57], and time pattern reconstruction [58] can help account for low to moderate missingness. Equally important is the design of transparent data collection systems that inform the study participant about the scientific need for specific sensor data collection, its intended usage, storage, and potential safety and privacy risks at the time of consent [59,60].

Digital health studies collecting data from multiple sensors [61] may suffer from significant analytical bias due to technical variations within and across devices that could potentially impact the accuracy and reproducibility of study outcomes. In the present dataset, the GPS data gathered exhibited high data missingness among all three sensors. Our findings corroborate with previous studies showing a higher GPS missingness across sensors [26]. In addition, we computed data missingness as a function of discontinuity in data sampling rather than overall counts of data records during an observation period (Table 3). As such, it is technically feasible for devices to have higher GPS data collection rates. However, subsampling could still significantly impact the data gathered per observation, leading to higher missingness in the output signal.

Furthermore, our results suggest that gyroscope sensor data can have significantly higher noise levels than accelerometer and GPS data, as evidenced by a lower signal-to-noise ratio (SNR) across device types. Such inter-sensor level differences could impact the development of robust and generalizable digital endpoints. For example, developing stratified digital markers of physical activity levels in the real world using multimodal sensors (e.g., GPS, accelerometer, and gyroscope) [62] could significantly impact inter-sensor data quality differences.

Our findings are based on smartphone-based sensor data collected in a large, fully remote decentralized study and should be viewed in the context of certain limitations of RWD collection. Although the cohort size for the present study is large and well-balanced across devices, we did not perform sensor data quality analysis on the entire WASH study cohort, mainly due to the data volume and download throughput limitations. The present analysis was also restricted to a subset of the most common sensors across Android and iOS: the accelerometer, gyroscope, and GPS. Moreover, the present study only compared cohort-level (i.e., cross-sectional) median DQM differences, although the analysis of the temporal (i.e., longitudinal) aspect of data quality would have helped identify more subtle differences. Future studies could use and extend our methods to assess the data quality of diverse smartphone sensors. Additionally, the data collection and consistency could be affected by participant engagement with the study app and the passive data-sharing settings (e.g., participants turning passive data sharing on/off). These user-specific settings and options participation incentives offered in studies collecting remote data could have impacted the interpretability and significance of the missing data metric (MDR) the most. In addition, high MDR values computed for GPS could be because GPS sampling follows an irregular on/off cyclic operational protocol depending on the device’s battery level at the time of collection, network coverage, and location detectability. Finally, the present inter-device comparison did not account for heterogeneity within the iOS and Android ecosystems due to a lack of available metadata. For example, variations in device models and operating system versions could further impact the sensor data collection and quality and should be further researched.

## 5. Conclusions

Our findings highlight the potential of significant differences in quality of sensor data collected from a large population using iOS and Android devices in real-world settings. We used eight data-driven quality metrics across three dimensions—completeness, correctness, and consistency. Our study demonstrated that there are differences in data quality both between different sensors and within the same sensor, including issues with missing data, noise contamination, and sampling inconsistencies across iOS and Android devices.

The use of sensor-based measurements of digital behavior in real-world settings holds broad promise—from aiding assessment of clinical efficacy of investigational drugs to learning individualized patient health-related behavior. However, there is a critical need to create robust and reliable data quality control standards in order for sensor-based RWD to accelerate patient-centered clinical research and care.

## Figures and Tables

**Table 1:** Data quality metrics’ description and mathematical definitions.

Data Quality Metric	Description	Example
Completeness		
Interpretable record length ratio (IRLR)	Ratio of records having 2 or more data points by the total number of records	IRLR of 0.8 results when 2 data records out of 10 have less than 2 points of length
Sensor channel ratio (SCR)	Ratio of recorded sensor channels by the typical number of channels	SCR of 0.66 results when an accelerometer record outputs 2 valid data channels instead of 3.
Missing data ratio (MDR)	Ratio of missing data samples by the expected total number of samples	MDR of 0.9 is obtained when 9 data points are collected, but 10 data points are expected to be generated with consistent data sampling
Correctness		
Signal-to-noise ratio (SNR)	Ratio of the expected signal amplitude by the noise amplitude	Positive SNR indicates that the amplitude of desired data is greater than that of undesired noise data
Anomalous point density (APD)	Ratio of anomalous data samples by the total number of samples	APD of 0.02 indicates that 2 out of 100 collected data points are outliers
Consistency		
Record length consistency (RLC)	Uniformity level of data record length within and across sensors	RLC of 1 indicates that a set of data records have the same length
Sampling rate consistency (SRC)	Uniformity level of data sampling rate within and across sensors	SRC of 0.9 indicates highly regular data sampling w.r.t. the typical data-driven sampling intervals
Value range consistency (VRC)	Uniformity level of data value range within and across sensors	VRC of 0 indicates high inter-record value range variability and instability

**Table 2:** Characteristics of the selected study cohort along with device-specific distribution.

	Selected Cohort N = 3000 *n (%)*	Android Users N = 1500 n (%)	iOS Users N = 1500 n (%)	Android vs. iOS (*p*-Value)
***Age (years)***				*p* < 0.05
19–29	1039 (49.7)	422 (42.5)	617 (56.1)	
30–39	529 (25.3)	283 (28.5)	246 (22.4)	
40–49	264 (12.6)	159 (16.0)	105 (9.6)	
50–59	156 (7.5)	78 (7.9)	78 (7.1)	
60+	103 (4.9)	50 (5.0)	53 (4.8)	
Invalid or missing	909	508	401	
***Gender***				*p* < 0.001
Female	1226 (53.7)	501 (43.1)	725 (64.7)	
Male	1057 (46.3)	662 (56.9)	395 (35.3)	
Invalid or missing	717	337	380	
***Race***				*p* < 0.001
White	1204 (51.8)	525 (44.2)	679 (59.8)	
Asian	365 (15.7)	121 (10.2)	244 (21.5)	
Hispanic	316 (13.6)	202 (17.0)	114 (10.0)	
Black or African-American	286 (12.3)	215 (18.1)	71 (6.2)	
Other	152 (6.5)	124 (10.4)	28 (2.5)	
Invalid or missing	677	313	364	
***Marital Status***				*p* = 0.13
Single	1167 (50.2)	494 (41.7)	673 (59.2)	
Married/Domestic partner	909 (39.1)	505 (42.6)	404 (35.5)	
Divorced	192 (8.3)	149 (12.6)	43 (3.8)	
Other	55 (2.4)	38 (3.2)	17 (1.5)	
Invalid or missing	677	314	363	
***Income Level***				*p* < 0.05
Less than $25,000	616 (29.5)	237 (24.5)	379 (33.7)	
$25,000 to $49,999	461 (22.1)	212 (21.9)	249 (22.2)	
More than $100,000	445 (21.3)	264 (27.3)	181 (16.1)	
$50,000 to $74,999	312 (14.9)	122 (12.6)	190 (16.9)	
$75,000 to $99,999	256 (12.2)	132 (13.7)	124 (11.0)	
Invalid or missing	910	533	377	
***Education***				*p* < 0.001
College	1258 (54.2)	529 (44.7)	729 (64.1)	
Graduate school	669 (28.8)	347 (29.3)	322 (28.3)	
High school and lower	395 (17.0)	308 (26.0)	87 (7.6)	
Invalid or missing	678	316	362	

The proportions are based on the number of participants who completed the baseline survey. Missing and invalid data were not used in the comparative analysis (see Appendix A).

**Table 3:** Device type classification using combined and specific sensor data quality metrics.

Metric	Combined Median [95% CI]	Accelerometer Median [95% CI]	Gyroscope Median [95% CI]	GPS Median [95% CI]
Accuracy	0.906 [0.905, 0.907]	0.947 [0.941, 0.954]	0.979 [0.977, 0.982]	0.868 [0.861, 0.872]
Precision	0.836 [0.808, 0.865]	0.767 [0.745, 0.789]	0.808 [0.774, 0.843]	0.782 [0.738, 0.825]
Recall	0.671 [0.619, 0.723]	0.769 [0.738, 0.798]	0.743 [0.691, 0.795]	0.627 [0.511, 0.743]
AU-PRC	0.939 [0.939, 0.940]	0.946 [0.946, 0.947]	0.968 [0.961, 0.972]	0.879 [0.874, 0.883]
Sensitivity	0.783 [0.717, 0.850]	0.806 [0.707, 0.905]	0.801 [0.663, 0.940]	0.553 [0.322, 0.784]
Specificity	0.829 [0.764, 0.894]	0.793 [0.707, 0.879]	0.581 [0.420, 0.742]	0.804 [0.621, 0.987]
AU-ROC	0.958 [0.956, 0.958]	0.945 [0.943, 0.945]	0.974 [0.973, 0.975]	0.958 [0.956, 0.960]

## Data Availability

The data supporting this study’s findings are available upon reasonable request from the Texas Advanced Computing Center, University of Texas at Austin, and will be made available to researchers upon request. Please contact Justin Drake at jdrake@tacc.utexas.edu. The data are not publicly available as they contain information that could compromise the privacy of research participants.

## Supplementary Materials

The following supporting information can be downloaded at: https://www.mdpi.com/article/10.3390/s24196246/s1, Figure S1: Comparison of data quality metrics per device type across race subgroups; Figure S2: Overall and sensor-specific device type classification receiver operator curves (ROC). Mean ROC bounded by 95% CI for 1000 stratified 12-fold cross-validation permutations; Figure S3: Boxplot of the effect of label permutation (N = 1000) on device type classification precision; Figure S4: Device-specific comparison of data quality metrics’ distributions across common sensors: accelerometer, gyroscope, and location; Figure S5: Sensor-specific comparison of data quality metrics’ distributions across devices: Android and iOS; Table S1: Base and composite sensor list per device type; Table S2: Characteristics of the full study cohort and the selected cohort; Table S3: Android sensor data quality metrics’ association with the study participants’ sociodemographics represented as pairwise effect size ε² and statistical significance; Table S4: iPhone sensor data quality metrics’ association with the study participants’ sociodemographics represented as pairwise effect size ε² and statistical significance; Table S5: Data quality metrics’ descriptive statistics across devices and sensors; Table S6: Descriptive statistics and significance of data quality differences across sensors with pairwise statistical significance; Table S7: Summary DQM count distribution per device and sensor type.

## Appendix A

The app allowed the users to submit their responses multiple times for the same survey, thereby generating duplicates and contradictory information. To mitigate this issue, we selected the last submitted entry per user as the final and accurate input.
