# Supplementary material for: Comparative Assessment of Multimodal Sensor Data Quality Collected Using Android and iOS Smartphones in Real-World Settings
Source: Sensors (Basel). 2024 Sep 26;24(19):6246. doi: 10.3390/s24196246 (PMC11478693; doi:10.3390/s24196246)

Table S1. Base and composite sensor list per device type.

		Android (N=21)	iPhone (N=7)
Base Sensors	Accelerometer	✓	✓
	Gyroscope	✓	✓
	Magnetometer	✓	✓
	Light	✓	✓
	Proximity	✓	✓
	Pressure	✓	✓
Composite Sensors	Game Rotation Vector	✓	✕
	Geomagnetic rotation vector	✓	✕
	Glance gesture	✓	✕
	Gravity	✓	✕
	Gyroscope uncalibrated	✓	✕
	Linear acceleration	✓	✕
	Magnetic field uncalibrated	✓	✕
	Orientation	✓	✕
	Pick up gesture	✓	✕
	Rotation vector	✓	✕
	Significant motion	✓	✕

	Step counter	✓	✓
	Step detector	✓	✕
	Tilt detector	✓	✕
	Wake up gesture	✓	✕

Table S2. Characteristics of the full study cohort and the selected cohort.

	Full study cohort<br>N = 10768<br>*n (%) or Median (IQR)*	Selected cohort<br>N = 3000<br>*n (%) or Median (IQR)*
**Age (years)**		
19-29	2949 (47.1)	1039 (49.7)
30-39	1637 (26.1)	529 (25.3)
40-49	804 (12.8)	264 (12.6)
50-59	490 (7.8)	156 (7.5)
60+	387 (6.2)	103 (4.9)
**Gender**		
Female	3817 (58.1)	1226 (53.7)
Male	2757 (41.9)	1057 (46.3)
**Race**		
Asian	931 (13.9)	365 (15.7)
Black or African American	723 (10.8)	286 (12.3)
Hispanic	783 (11.7)	316 (13.6)
Other	306 (4.6)	152 (6.5)
White	3938 (58.9)	1204 (51.8)
**Marital Status**		
Divorced	410 (6.1)	214 (8.5)
Married/Domestic Partner	2821 (42.2)	966 (38.4)
Single	3312 (49.6)	1278 (50.8)
Other	138 (2.1)	60 (2.3)
**Income Level**		
Less than $25,000	1736 (30.0)	660 (29.0)
$25,000 to $49,999	1268 (21.9)	497 (21.8)
$50,000 to $74,999	886 (15.3)	352 (15.5)

$75,000 to $99,999	710 (12.3)	271 (11.9)
More than $100,000	1193 (20.6)	495 (21.7)
**Education**		
High School and lower	868 (13.0)	422 (16.7)
College	3881 (58.1)	1356 (53.9)
Graduate School	1928 (28.9)	740 (29.4)

Table S3. Android sensor data quality metrics' association with the study participants' socio-demographics represented as pairwise effect size ε^2 and statistical significance.

. p< .05
* p < .01
** p< .001
*** p< .0001

	Age	Gender	Race	Marital Status	Income Level	Education
IRLR	0.002	0.001	0.002	0.006	0.007	0.004
SCR	1	1	1	1	1	1
MDR	0.029**	0.004·	0.126***	0.014·	0.033***	0.008
SNR	0.025	0.015	0.080	0.028	0.019	0.008
APD	0.009	0	0.004	0.008	0.008	0.003
RLC	0.001**	0.001**	0.010***	0.003**	0.001*	0.002·
SRC	0.046***	0.006·	0.115***	0.018*	0.030***	0.001
VRC	0.019*	0.002	0.085***	0.008	0.053***	0.004

Table S4. iPhone sensor data quality metrics' association with the study participants' socio-demographics represented as pairwise effect size ε² and statistical significance.

. p< .05
* p < .01
** p< .001
*** p< .0001

	Age	Gender	Race	Marital Status	Income Level	Education
IRLR	0.015	0.01	0.004	0.008	0.003	0.002
SCR	1	1	1	1	1	1
MDR	0.007**	0.	0.007***	0.005.	0.005***	0.009
SNR	0.006	0	0.003	0.002	0.002	0.005
APD	0.001	0	0.013	0.007	0.003	0.001
RLC	0.005**	0**	0.022***	0.022**	0.004*	0.003.
SRC	0.006	0.001.	0.008	0.008.	0.011.	0.009.
VRC	0.001	0	0.003	0.007	0.002	0.005

Table S5. Data quality metrics descriptive statistics across devices and sensors.

	Device			Sensor			
	Android *Median(IQR)*	iOS *Median(IQR)*	p-value	Accel *Median(IQR)*	Gyro *Median(IQR)*	GPS *Median(IQR)*	p-value
Completeness							
Missing Data Ratio (MDR)	0.39(0.38)	0.16(0.36)	p < 0.0001	0.22(0.37)	0.23(0.37)	0.51(0.62)	p < 0.0001
Sensor Channel Ratio (SCR)	1(0)	1(0)	1	1(0)	1(0)	1(0)	1
Interpretable Record Length Ratio (IRLR)	1(0.01)	1(0)	p < 0.0001	1(0)	1(0)	1(0.1)	p < 0.0001
Correctness							
Anomalous Point Density (APD)	0.02(0.01)	0.01(0.01)	p < 0.0001	0.01(0.01)	0.01(0.01)	0.01(0.01)	p < 0.0001
Signal-to-noise Ratio (SNR)	-3.01(3.77)	-5.73(8.86)	p < 0.0001	-3.11(1.38)	-9.74(5.70)	-2.52(1.45)	p < 0.0001
Consistency							
Sampling Rate Consistency (SRC)	0.35(0.35)	0.42(0.42)	p < 0.0001	0.42(0.40)	0.40(0.43)	0.30(0.27)	p < 0.0001
Record Length Consistency (RLC)	0.54(0.26)	0.56(0.18)	p < 0.0001	0.55(0.21)	0.54(0.19)	0.56(0.36)	p<0.01
Value Range Consistency (VRC)	0.61(0.35)	0.73(0.16)	p < 0.0001	0.69(0.19)	0.70(0.20)	0.49(0.61)	p < 0.0001

Table S6. Descriptive statistics and significance of data quality differences across sensors with pairwise statistical significance.

	Sensor			p-value		
	Accel Median(IQR)	Gyro Median(IQR)	GPS Median(IQR)	Accel vs. Gyro	Accel vs. GPS	Gyro vs. GPS
**Completeness**						
Missing Data Ratio (MDR)	0.22(0.37)	0.23(0.37)	0.51(0.62)	$p > 0.05$	$p < 0.0001$	$p < 0.0001$
Sensor Channel Ratio (SCR)	1(0)	1(0)	1(0)	$p < 0.05$	$p < 0.0001$	$p < 0.0001$
Interpretable Record Length Ratio (IRLR)	1(0)	1(0)	1(0.1)	$p < 0.05$	$p < 0.0001$	$p < 0.0001$
**Correctness**						
Anomalous Point Density (APD)	0.01(0.01)	0.01(0.01)	0.01(0.01)	$p < 0.05$	$p < 0.0001$	$p < 0.0001$
Signal-to-noise Ratio (SNR)	-3.11(1.38)	-9.74(5.70)	-2.52(1.45)	$p > 0.05$	$p < 0.0001$	$p < 0.0001$
**Consistency**						
Sampling Rate Consistency (SRC)	0.42(0.40)	0.40(0.43)	0.30(0.27)	$p > 0.05$	$p < 0.0001$	$p < 0.0001$
Record Length Consistency (RLC)	0.55(0.21)	0.54(0.19)	0.56(0.36)	$p < 0.05$	$p > 0.05$	$p > 0.05$
Value Range Consistency (VRC)	0.69(0.19)	0.70(0.20)	0.49(0.61)	$p > 0.05$	$p < 0.0001$	$p < 0.0001$

Table S7. Summary DQM count distribution per device and sensor type.

	Android<br>N = 3585<br>*n*	iOS<br>N = 2331<br>*n*
**Sensor**		
Accelerometer	1377	1013
Gyroscope	1032	939
GPS	1176	379

Figure S1**.** Comparison of data quality metrics per device type across race subgroups.

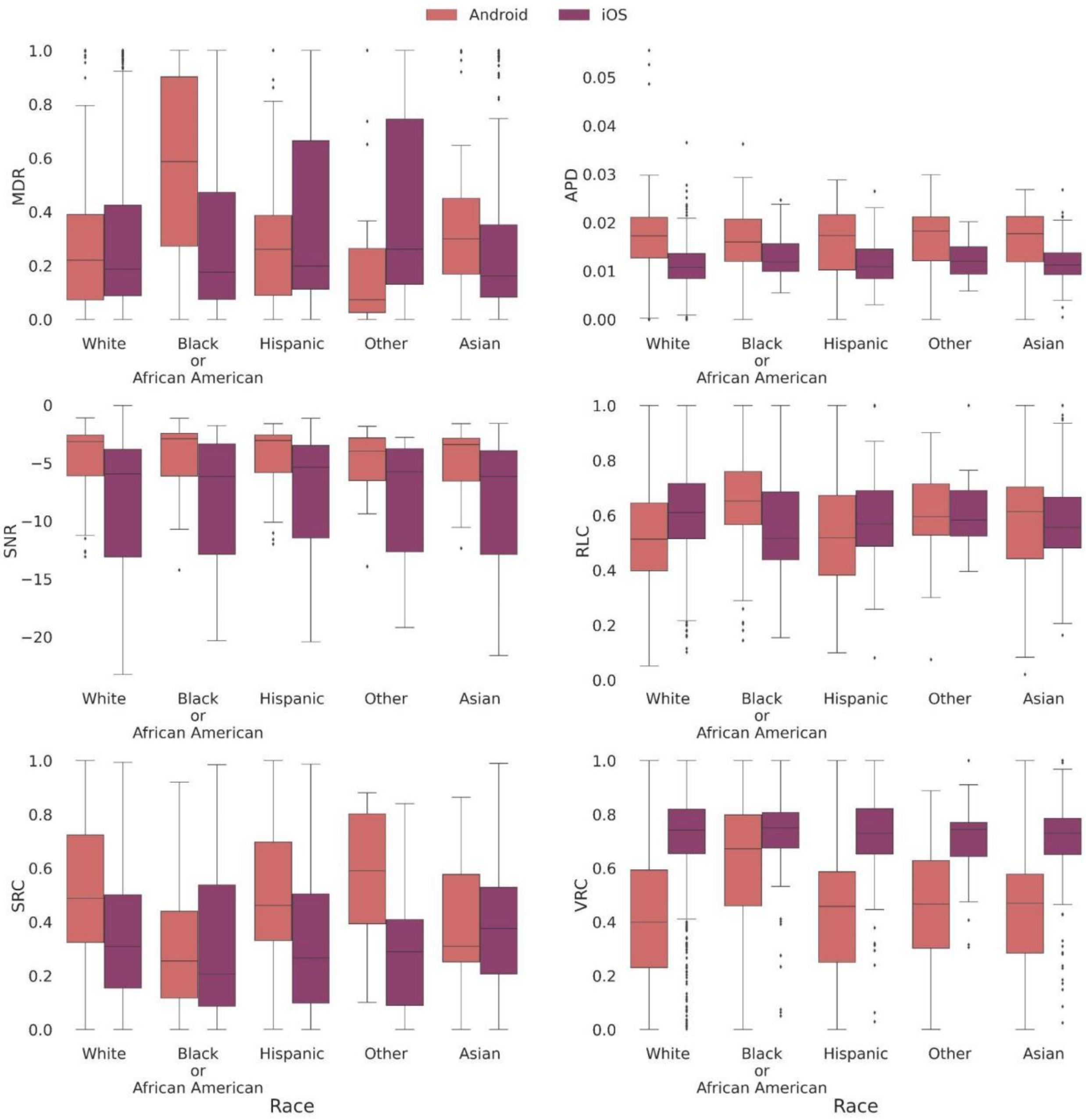

Figure S2. Overall and sensor-specific device type classification receiver operator curves (ROC). Mean ROC bounded by 95% CI for 1000 stratified 12-fold cross-validation permutations.

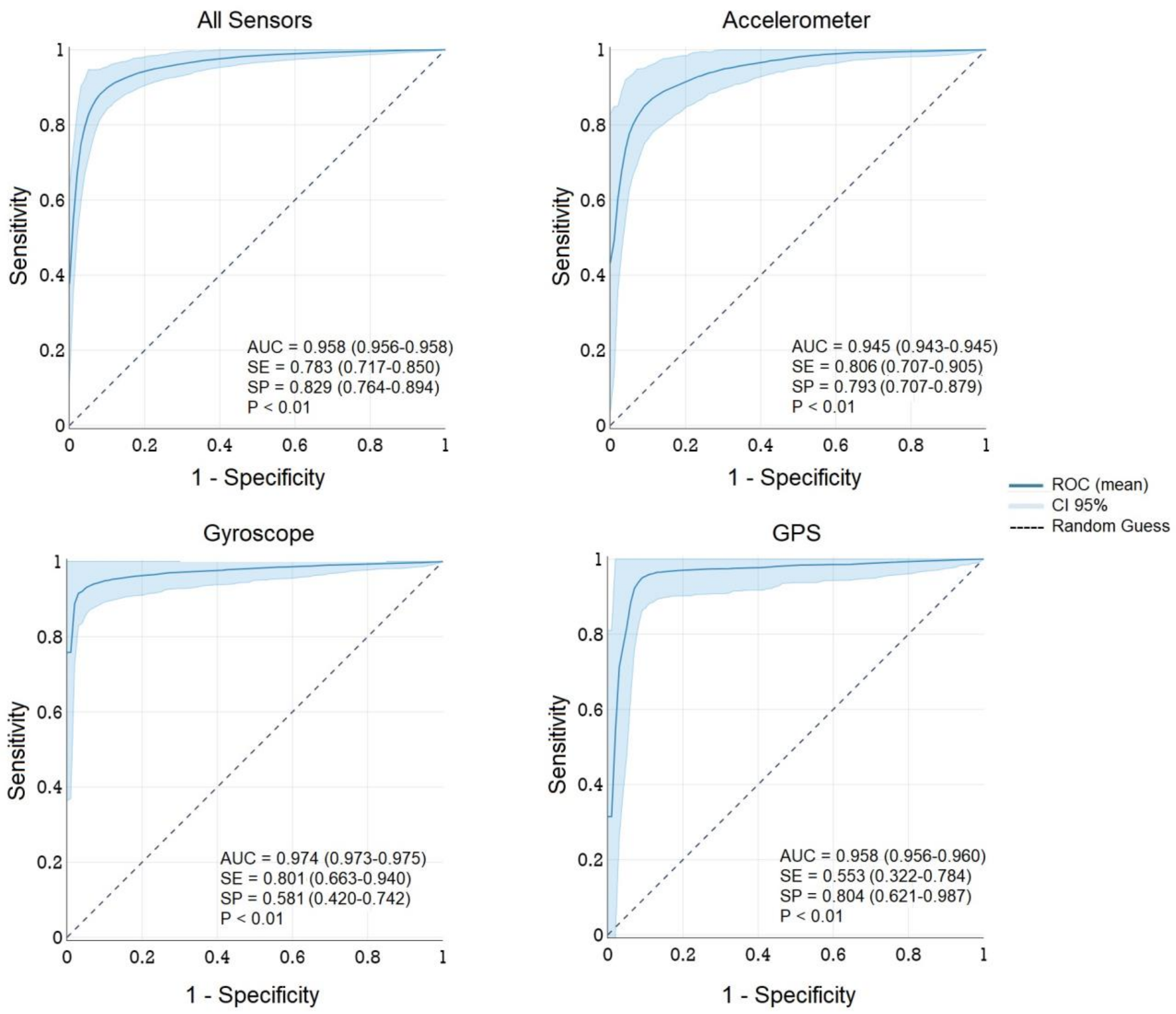

Figure S3**.** Boxplot of the effect of label permutation (N=1000) on device type classification precision.

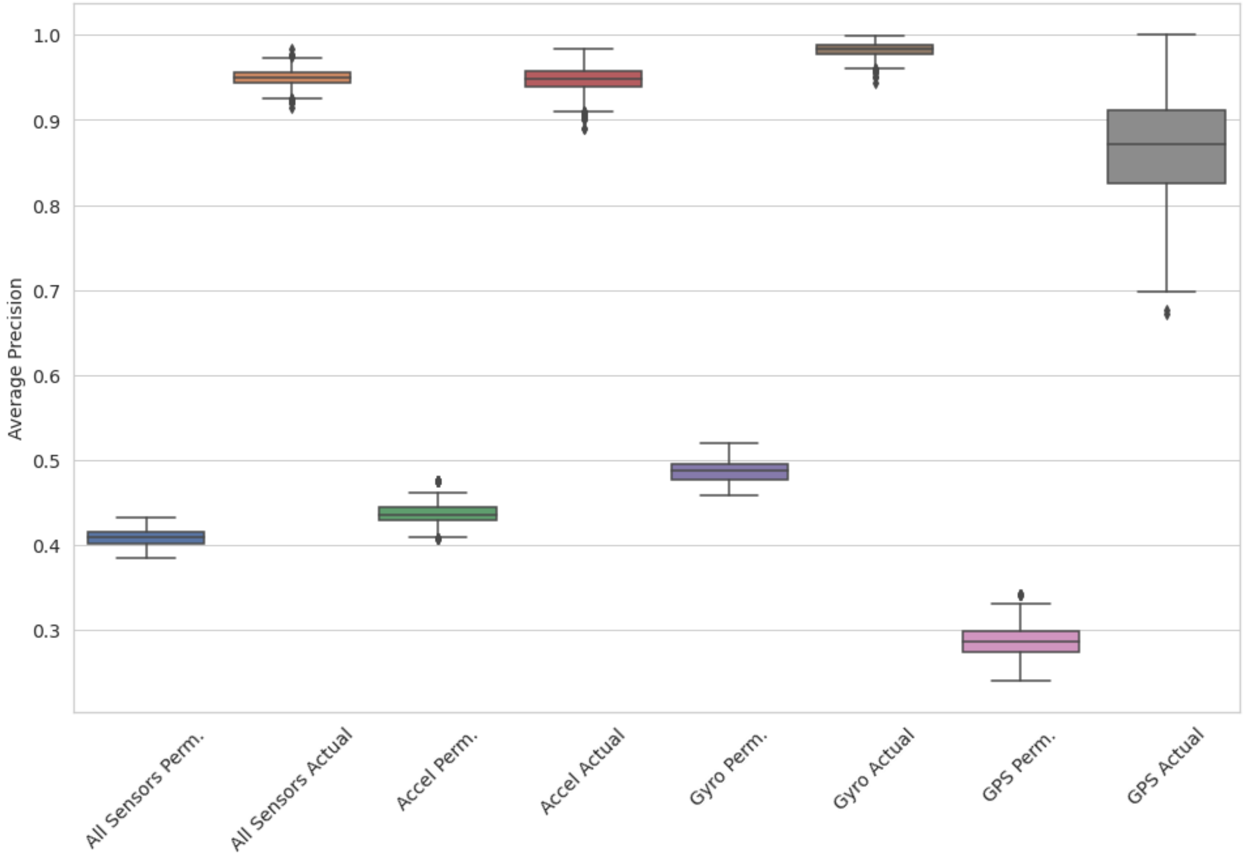

Figure S4. Device-specific comparison of data quality metrics' distributions across common sensors: accelerometer, gyroscope, and location.

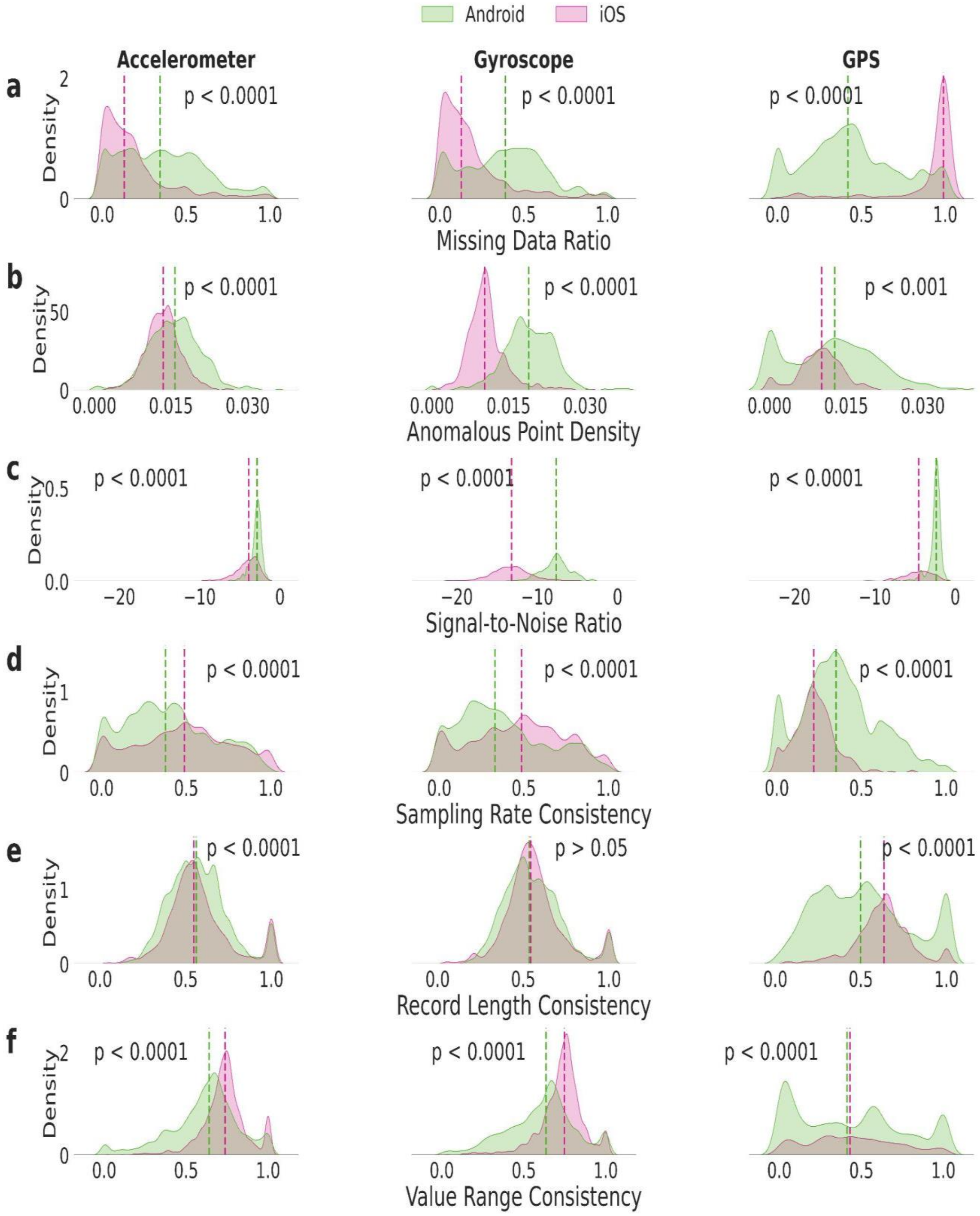

Figure S5**.** Sensor-specific comparison of data quality metrics' distributions across devices: Android and iOS.

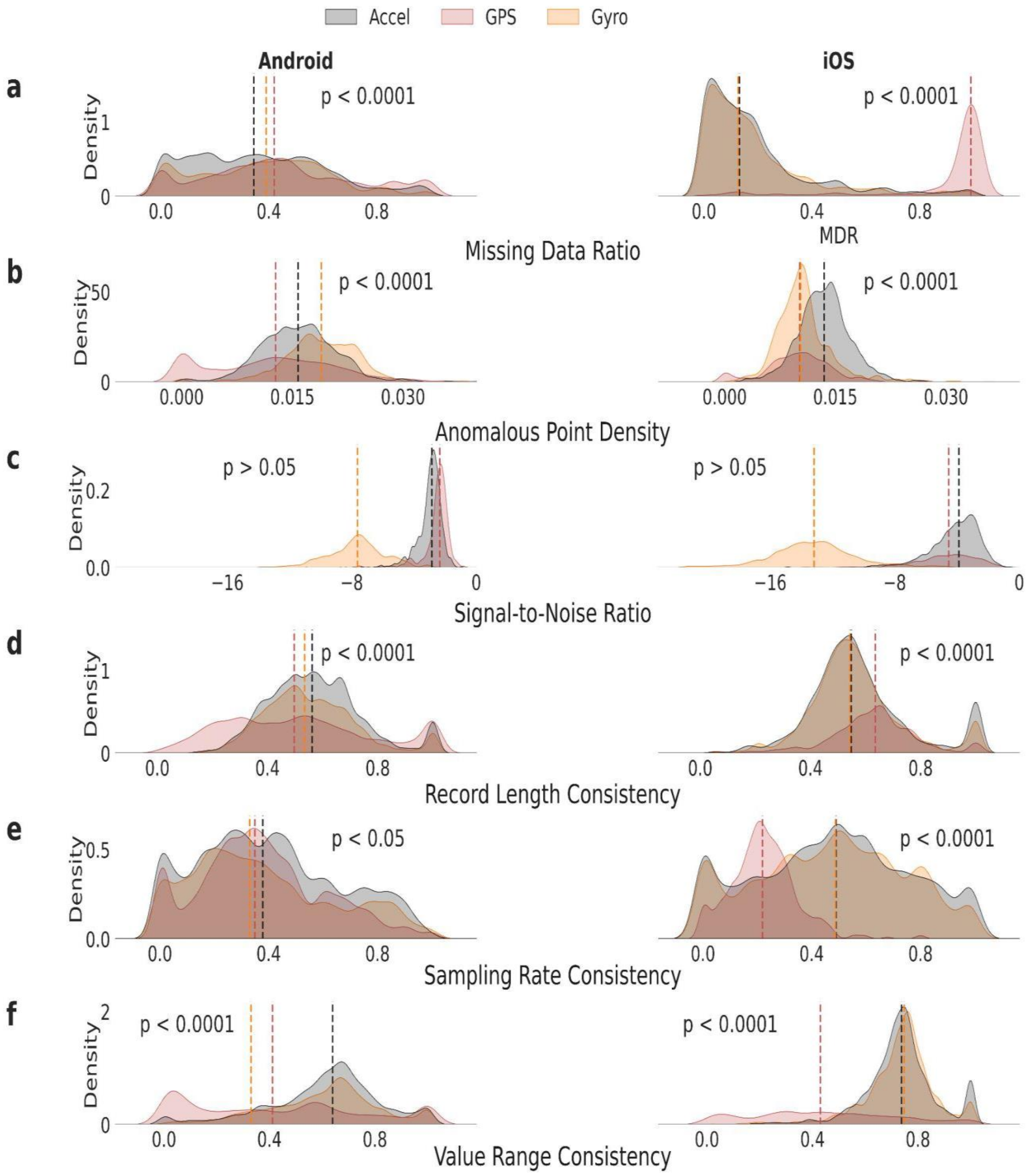

Supplement: Supplementary file 1 [file sensors-24-06246-s001.zip › sensors-3102159-supplementary.pdf]
